# The utility of liquid biopsy in clinical genetic diagnosis of cancer and monogenic mosaic disorders

**DOI:** 10.1515/medgen-2023-2066

**Published:** 2023-12-05

**Authors:** Ariane Hallermayr, Thomas Keßler, Verena Steinke-Lange, Ellen Heitzer, Elke Holinski-Feder, Michael Speicher

**Affiliations:** MGZ – Medizinisch Genetisches Zentrum Munich Germany; MGZ – Medizinisch Genetisches Zentrum München Germany; MGZ – Medizinisch Genetisches Zentrum Bayerstraße 3–5 80335 München Germany; Medical University of Graz Institute of Human Genetics, Diagnostic and Research Center for Molecular Biomedicine (Austria) Graz Austria; MGZ – Medizinisch Genetisches Zentrum München Germany; Medical University of Graz Institute of Human Genetics, Diagnostic and Research Center for Molecular Biomedicine (Austria), Neue Stiftingtalstraße 2 Graz Austria

**Keywords:** liquid biopsy, cancer diagnostics, human genetics, clonal hematopoiesis, mosaic disorders

## Abstract

Liquid biopsy for minimally invasive diagnosis and monitoring of cancer patients is progressing toward routine clinical practice. With the implementation of highly sensitive next-generation sequencing (NGS) based assays for the analysis of cfDNA, however, consideration of the utility of liquid biopsy for clinical genetic testing is critical. While the focus of liquid biopsy for cancer diagnosis is the detection of circulating tumor DNA (ctDNA) as a fraction of total cell-free DNA (cfDNA), cfDNA analysis reveals both somatic mosaic tumor and germline variants and clonal hematopoiesis. Here we outline advantages and limitations of mosaic and germline variant detection as well as the impact of clonal hematopoiesis on liquid biopsy in cancer diagnosis. We also evaluate the potential of cfDNA analysis for the molecular diagnosis of monogenic mosaic disorders.

## Background

**Figure 1:**
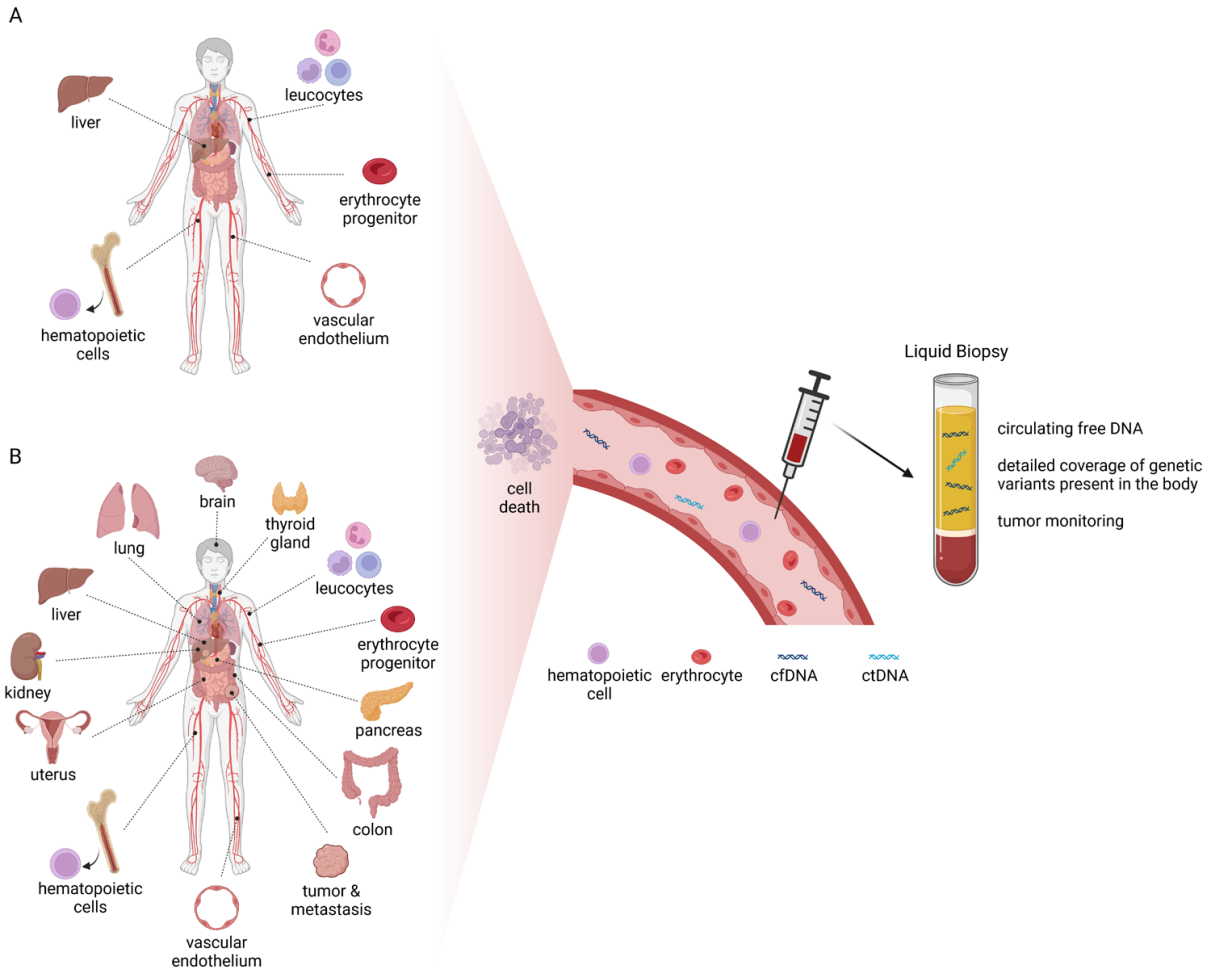
cfDNA is released upon cell death into circulation. (A) In healthy individuals, cfDNA is released mainly from leukocytes, vascular endothelium, erythrocyte progenitor cells, bone marrow and hepatocytes. (B) In the case of diseases such as cancer or mosaic disorders, it is possible that cfDNA is released from affected tissues. (cfDNA: cell-free DNA; ctDNA: circulating tumor DNA) [65] (created with BioRender.com)

Among other analytes, liquid biopsy enables the analysis of circulating nucleic acids, such as cell-free DNA (cfDNA) from blood plasma and other body fluids. In blood, cfDNA mainly originates from apoptosis of hematopoietic cells, but other cell types additionally can contribute to the overall cfDNA pool (Figure 1) [1]. In cancer patients also tumor sites release cfDNA, that carries different types of tumor-specific genetic alterations and is referred to as circulating tumor DNA (ctDNA) [2]. Due to the typical fragmentation patterns during apoptosis, cfDNA presents a modal size of 167 bp, which corresponds to the length of DNA bound by one nucleosome plus linker DNA [3].

Moreover, biological properties of cfDNA, such as fragmentation profiles or the cfDNA levels from different tissues of origin, differ depending on the body fluid. The fragment length of cfDNA from urine is, for example, shorter (<100 bp) than the fragment length of plasma cfDNA with the nucleosome pattern being less pronounced. Furthermore, for brain tumors the fraction of tumor-derived fragments has been demonstrated to be higher in cerebrospinal fluid (CSF) compared to plasma, most likely due to the blood-brain barrier and the fact that CSF contains fewer immune cells, thus having a lower proportion of non-tumor DNA. Therefore, proximal sampling, i. e., analysis of tumor DNA from sources closer to the tumor compared with those from peripheral veins might be preferrable- such as CSF for tumors of the central nervous system, or urine for urogenital cancers – and might achieve higher sensitivities to address the respective clinical question [4].

Liquid biopsy is emerging as a promising tool with minimal invasiveness for clinical diagnostics of various diseases. These applications of liquid biopsy for patients with cancer currently include:

– companion diagnostics (CDx) to guide treatment decisions [5],
– detection of minimal residual disease (MRD) post-surgery [6],
– real-time monitoring to predict response or resistance to treatment [7],
– early detection of cancer in seemingly healthy individuals [8].

These applications require sensitive methods to detect somatic variants with very low variant allele frequencies (VAFs). The highest sensitivities traditionally have been achieved with methods such as digital PCR (dPCR), enabling the detection of single variants with VAFs from 0.1 %. Recent advances in next-generation sequencing (NGS) such as strand-aware barcoding of DNA fragments, improved the sensitivity and specificity of NGS assays, enabling the detection of multiple variants in parallel in a comparabale range of VAFs [9, 10]. While NGS comes with higher cost, analysis of broader genomic regions from a single blood sample rather than focusing on specific hotspots represents an advantage for CDx. In addition to detecting informative variants for CDx, liquid biopsy also holds great potential for disease surveillance or cancer screening. Since not all cancers carry hotspot variants, current research is focused on the untargeted detection of ctDNA. Here, cfDNA fragmentomics and epigenetic signatures are of particular interest. In cfDNA fragmentomics cfDNA fragment lengths and sequencing depth are analyzed as these indirectly represent the chromatin structure and therefore enable the highly sensitive detection of ctDNA and even the indirect evaluation of gene expression [11, 12, 13, 14]. Furthermore, DNA methylation analysis has been described as a promising tool in cancer screening [8]. However, such approaches are still within the scope of research and not yet applied in clinical practice.

Liquid biopsies are applied in a growing number of non-cancer-related applications. The best-established applications are non-invasive prenatal testing (NIPT), specifically analyzing cell-free fetal DNA (cffDNA) to identify potential genetic variants distinct to the fetus [15]. This topic is reviewed in another article of this issue and will not be further discussed here. As established and novel liquid biopsies methods more and more come into focus of everyday diagnostic application, the relevance and potential pitfalls of liquid biopsy methods in the context of human genetics increases as well. For example, an increasing use of NGS based ctDNA testing to identify variants in multiple cancer genes simultaneously, increases the chance that germline variants with far-reaching consequences for the patient and their family may be identified in liquid biopsy analyses, highlight the high relevance of human genetics specialists in the process of variant interpretation and reporting. In the following, we focus on a brief discussion of four main aspects, i. e., the detection of hotspot variants in cancer, mosaicism, clonal hematopoiesis, and germline variants in cfDNA.

**Figure 2:**
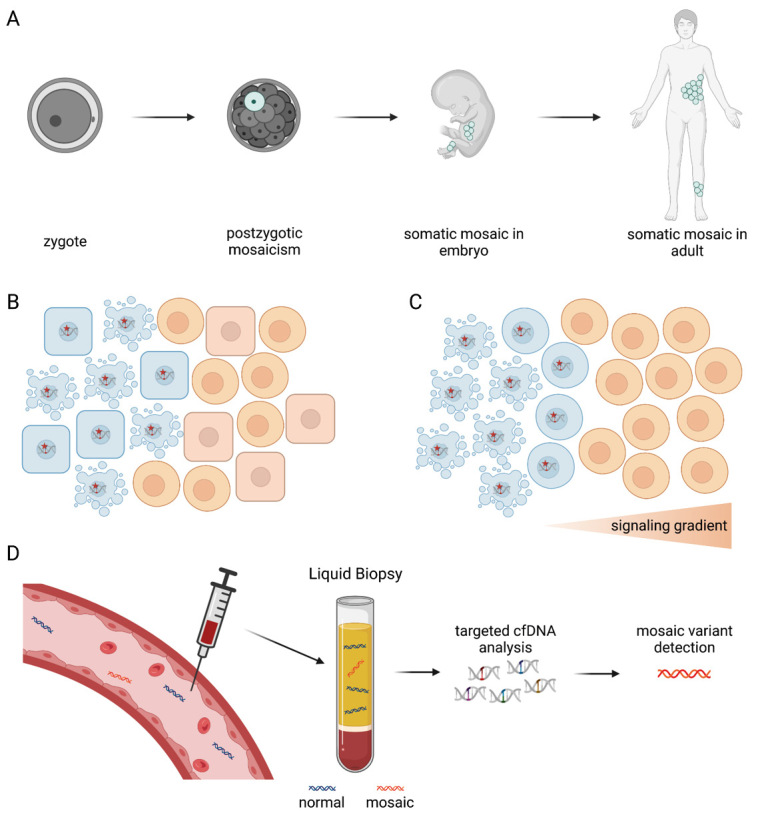
(A) Variants arising from postzygotic mosaicism may be present as mosaic variants in adults. Although somatic mosaicism is suspected, a tissue biopsy might reveal only low VAFs of the somatic variant. This could be caused by (B) cell type-specific lethality or (C) impaired paracrine or juxtacrine signaling. (D) Performing liquid biopsy analysis can increase the diagnostic yield of the molecular cause of mosaic disorders by detecting mosaic variants within total cfDNA. [65] (created with BioRender.com)

## Liquid biopsy for the detection of hotspot variants in cancer

As described above liquid biopsy holds great potential for companion diagnostics and disease monitoring in cancer. During cancer pathogenesis certain genes are frequently mutated across different cancer types. Among the numerous genetic alterations, hotspot variants in tumor suppressor genes are frequently observed. While inactivating variants may distribute along the coding sequence of tumor suppressor genes such as *TP53*, variants in proto-oncogenes frequently hit distinct hotspot-regions, such as common activating variants in codon 12 and 13 of *KRAS* [16, 17]. While *TP53* and *KRAS* are among the most frequently altered genes across cancer types, distinct hotspot variants are specific to distinct cancer types, such as activating *EGFR* variants in exons 18 to 21 in non-small cell lung cancer (NSCLC)[64] or *PIK3CA* variants in codons 542, 545 and 1047 in breast cancer [18]. Such oncogenic variants occur due to errors during DNA replication and drive cancer growth. Modern treatment options include drugs specifically targeting such variants, allowing personalized treatment interventions, such as anti-*EGFR* tyrosine kinase inhibitors (TKIs) [19]. However, a large number of patients develop resistance against those inhibitors throughout treatment, with the *EGFR* p.T790M mutation being the frequent cause of resistance in ~50 % of cases [20]. Therefore, tracking the appearance of the *EGFR* p.T790M variant during treatment may serve as a biomarker for a change in therapy towards third-generation *EGFR*-inhibitors, such as Osimertinib [21].

Traditionally, tissue biopsies are collected to evaluate tumor hotspot variants for companion diagnostics. However, the presence of ctDNA in total cfDNA also enables the detection of those variants within minimally invasive liquid biopsies. Correlation of ctDNA fraction with total tumor burden even enables monitoring of response or resistance to treatment by tracking ctDNA dynamics [7].

## Liquid biopsy for the detection of pathogenic variants in mosaic disease

**Figure 3:**
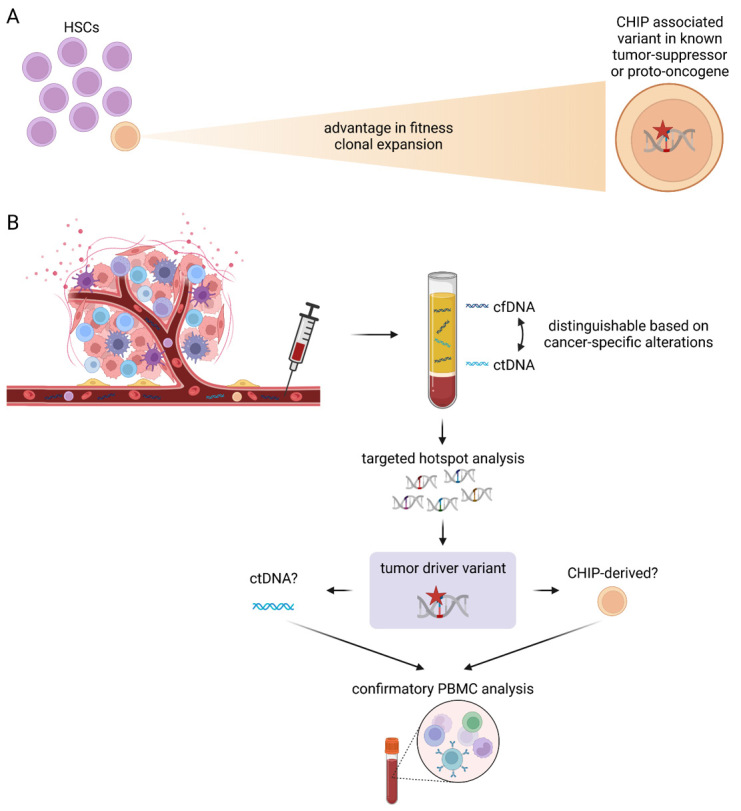
(A) Clonal hematopoiesis of indeterminate potential (CHIP) describes the clonal expansion of hematopoietic stem cells (HPCs) with acquired variants affecting known tumor suppressor or proto-oncogenes. (B) These CHIP-associated variants can be detected with highly sensitive liquid biopsy assays. To distinguish the presence of ctDNA from CHIP-derived variants, confirmatory peripheral blood mononuclear cell (PBMC) analysis may be required. [65] (created with BioRender.com)

Besides the potential of liquid biopsy in detecting and tracking hotspot variants in cancer, it also holds great potential for diagnosing non-cancer-related genetic diseases. Congenital genetic variants can either be inherited and are then present in all cells of the body or arise from postzygotic mosaicism resulting in the presence of such variants only in a subset of cells (mosaic variants) (Figure 3A). The number of cells affected by mosaicism greatly depends on when the mutation occurred [22]. Somatic variants occurring throughout development include different types of genetic changes, including single nucleotide variants (SNVs), copy number variants (CNVs), small insertions and deletions (InDels), structural variants (SVs) and repeat expansions [23]. Although somatic variants are often benign, somatic pathogenic variants can lead to monogenic diseases. Herein, the time of occurrence of somatic pathogenic variants during development is critical and ultimately determines the severity of the resulting phenotype, ranging from possible embryonic lethality to the expression of these variants in adult tissue(s) [24].

Various monogenic disorders caused by somatic mosaicism of pathogenic variants have been described and can be grouped into obligatory somatic mosaicism and nonobligatory somatic mosaicism. Obligatory somatic mosaicism is defined by lethality of a pathogenic variant during early developmental stages that prevents inheritance of the variant. Non-obligatory somatic mosaicism is characterized by presence of a heterozygous dominant pathogenic variant, that can be either inherited or appears de-novo as a postzygotic mosaicism. [22]. Several intracellular signaling pathways in cancer development are also affected in monogenic mosaic disorders such as neurocutaneous disorders or overgrowth syndromes [25]. For example, disruption of the PI3K/AKT pathway is associated with obligatory mosaic disorders, such as *PIK3CA*-related overgrowth spectrum (PROS) caused by somatic pathogenic variants in *PIK3CA*. These disorders include congenital lipomatous overgrowth, vascular malformations, epidermal nevi, skeletal anomalies (CLOVES, *PIK3CA* variants), megalencephaly-capillary malformation (MCAP, also caused by *PIK3CA* variants), Proteus syndrome (*AKT1* variants) and Smith-Kingsmore syndrome (*MTOR* variants). Somatic pathogenic variants in genes of the RAS/MAPK signaling pathway, in turn, are causative for RASopathies such as Noonan syndrome (*BRAF, MAP2K1, PTPN11,* or* RAF1* variants), Costello syndrome (*HRAS* variants) as well as other Noonan-like syndromes [26]. The two most common nonobligatory mosaic disorders are Neurofibromatosis Type-1, which can be caused by either inherited or somatic pathogenic variants in *NF1,* a negative regulator of the RAS/MAPK pathway [27], and mesial temporal lobe epilepsy caused by somatic variants in the same pathway [28. Besides variants in genes of the PI3K/AKT or RAS/MAPK pathways, somatic mosaicism in *GNAQ* or *GNAS* is associated with monogenic disorders such as Sturge-Weber syndrome (*GNAQ* variants) or McCune-Albright syndrome (*GNAS* variants) [29, 30].

To confirm somatic mosaicism as a cause of a suspected syndrome, a biopsy of the affected tissue is usually collected, and genomic DNA from this sample is analyzed. It is crucial to consider the purity of the tissue biopsy to achieve the highest sensitivity for somatic pathogenic variant detection above the background noise of the respective method. This is critical since low VAFs down to 1 % have been observed for various mosaic disorders in tissue biopsies [30]. Besides the “contamination” of a tissue biopsy with normal cells, other potential reasons for such low VAFs of somatic pathogenic variants is the occurrence of cell type-specific lethality or impaired paracrine or juxtacrine signaling, decreasing cell viability (Figure 3B and 3C) [23].

The difficulties implicated in the molecular analysis of mosaic disorders based on tissue biopsies might be overcome or amended by using liquid biopsy for diagnostic purposes, given the potential of liquid biopsy to detect genetic material from tissues in circulation. By analyzing cfDNA, therefore, not only inherited variants but also somatic variants associated with mosaic syndromes can be uncovered since decreased cell viability due to impaired paracrine or juxtacrine signaling or cell type-specific lethality might lead to an increased fraction of cfDNA released from mosaic cells (Figure 3D). As several liquid biopsy assays were explicitly developed for the detection of variants with <1 % VAF, the detection of low-level mosaicism is technically possible, as shown, for example, in patients with PROS or RASopathies [31], or in patients with somatic mosaicism in the brain leading to epilepsies [32] including via CSF liquid biopsies [33, 34]. Thus, adding liquid biopsy to the diagnostic toolbox can increase the diagnostic yield for patients with suspected mosaic disorders. As tissue biopsies are invasive measures that must be applied carefully, especially in children, and are rarely performed for certain tissues (like brain cells), liquid biopsies pave the way for novel diagnostic procedures.

With increasing interest in targeted therapies to treat mosaic disorders, a molecular diagnosis becomes more important to guide treatment decisions. Based on the identified somatic pathogenic variants, anti-cancer drugs such as *PIK3CA* or MEK inhibitors could be applied as therapy, i. e., *PIK3CA* inhibition is clinically effective without substantial side effects in patients with PROS [35]. Furthermore, a recent study using organoid and zebrafish models of central conducting lymphatic anomaly (CCLA) associated with overactivation of the RAS/MAPK pathway showed promising results to improve the phenotype by using MEK inhibitors, suggesting that MEK inhibition could be explored as a possible treatment option for CCLA-patients in future clinical trials [36].

Overall, liquid biopsy is a promising tool for diagnosing monogenic mosaic disorders. Because cfDNA is released from various tissues, including mosaic cells, detecting pathogenic variants from body fluids may improve the diagnostic yield of somatic mosaicism. In particular, the potential of targeted therapies to inhibit overactivated cell signaling pathways highlights the importance of identifying the molecular cause of such diseases.

## The Impact of clonal hematopoiesis on ctDNA specific variant detection

The number of somatic variants steadily increases throughout aging and an acquired advantage in the fitness of a single cell caused by such somatic variants can lead to clonal expansion of the respective cell [37]. The best-documented example of clonal expansion are hematopoietic stem cells (HSCs, clonal hematopoiesis) (Figure 2A) [38], but have by now also been reported in various epithelial tissues such as skin and esophagus [39]. Clonal hematopoiesis refers to all clonal expansion events in HSCs regardless of an individual’s phenotype. Besides malignant clonal hematopoiesis associated with cytopenias and dysplastic hematopoiesis and nonmalignant clonal hematopoiesis, there is also a third class called “clonal hematopoiesis of indeterminate potential” (CHIP), describing nonmalignant clonal hematopoiesis linked to variants frequently detected in cancers [40].

About 10 % of individuals >65 years of age present with CHIP [22]. CHIP has been identified as a risk factor for human diseases directly correlated to the size of the clone. For example, CHIP has been associated with an increased risk of developing hematologic malignancies since the “first hit” in malignancy transformation has already occurred [41]. Further, an association of CHIP with nonmalignant disorders has been described since immune cells are not locally restricted but can migrate to and influence almost all organs [42]. This effect of CHIP was shown to have implications on the risk of developing cardiovascular disease, chronic obstructive pulmonary disease, chronic liver disease, osteoporosis, and gout [43]. Thus, the biological processes underlying CHIP are relevant for our understanding of the aging processes in the hematological system per se and the modulation of other age-related health risks in individuals.

In recent years it has been shown that the clonal cell expansion that defines CHIP is caused by somatic chromosomal alterations, loss of sex chromosomes as well as acquired somatic variants in a distinct set of genes [44]. Among those genetic alterations, variants in *DNMT3A* and *TET2* are most common across all patients, indicating that DNA-methylation and chromatin accessibility are significant factors during CHIP-associated deregulation of HSC proliferation. Originally, CHIP variants were defined as variants in genes described to be affected in hematologic cancers that are present with a VAF of 2 % or higher [40]. A VAF of >2 % in peripheral blood mononuclear cells (PBMCs) means that at least 4 % of HSCs carry an assumed heterozygous variant. More recently, there is increasing evidence of CHIP variants detected in genes that are commonly mutated in solid tumors such as *TP53*, *NF1,* and *KRAS* [45]. Contrary to the hematological cancer genes (*DNMT3A*, *TET2*, and *ASXL1*) these variants are often detected at a much lower VAF (0.1–0.5 %) [46 and can give rise to a significant biological noise. Interestingly, genes involved in DNA damage repair pathways (e. g., *TP53)* appear more frequently affected by somatic variants in patients previously exposed to chemo- and/or radiotherapy [47].

Therefore, cfDNA derived from a CHIP-associated clone may be misinterpreted as ctDNA, particularly in elderly cancer patients with previously unrecognized CHIP [48]. This effect may pose a significant issue when highly sensitive ctDNA assays are used for cancer screening, tumor agnostic CDx to guide treatment decisions, or MRD detection without information about the patient’s tumor mutational status. In such cases, variants in known CHIP-associated genes should be interpreted with caution. Before clinical reporting, paired genotyping of a patient’s PBMCs is recommended to exclude a CHIP event and to prevent a misleading diagnosis (Figure 2B) [48]. With an increasing understanding of the biophysical and genomic features of ctDNA and CHIP, it might be possible to improve the interpretation of cfDNA variants further. The shorter fragment length of ctDNA might support the identification of tumor-derived variants [49]. Additionally, SNVs detected in CHIP are frequently C>T transitions, consistent with age-related signatures caused by spontaneous deamination of 5-methylcytosine [50]. Such age-related signatures are enriched in CHIP cfDNA fragments but absent in ctDNA fragments. The contrary effect has been observed for smoking-related DNA signatures, as those have been enriched in ctDNA fragments but absent in CHIP cfDNA fragments [51]. Recent studies even investigate the potential of specific, clonal hematopoiesis-derived signatures for cancer screening [52].

In conclusion, when interpreting results from a liquid biopsy, it is critical to confirm that the detected variant is specific to the tumor and does not represent a CHIP-derived variant. While it is currently recommended to ensure specific ctDNA detection by parallel analysis of PBMCs, in the future, it might be possible to differentiate ctDNA from CHIP cfDNA based on particular DNA signatures.

## Incidental findings of germline or somatic mosaic variants in cfDNA analysis

Comprehensive genomic profiling (CGP) for the detection of ctDNA in total cfDNA is increasingly being implemented into clinical decision-making and guiding treatment decisions. However, as with tissue profiling, in addition to somatic tumor-specific variants, also germline variants or mosaic variants e. g., in cancer predisposition genes originating from tissue cells other than blood cells may be detected as incidental findings. In fact, recent studies have demonstrated that in approximately 3–13 % of patients undergoing CGP from tumor tissue, pathogenic germline variants have been identified as incidental findings [53]. While germline variants may be present in higher VAFs than variants originating from ctDNA, constitutional mosaic variants are present in cfDNA at low VAFs, as described in the chapter on somatic mosaicism, and may therefore not be distinguished from ctDNA in cancer patients, with no previous knowledge of such a somatic mosaicism.

The American College of Medical Genetics and Genomics (ACMG) recommends reporting incidental findings of pathogenic germline variants in several genes, as these findings can have significant consequences not only for the patients but also for their healthy family members [54]. However, the origin of genetic variants detected in cfDNA analysis cannot be determined with certainty as either somatic or germline. Specific criteria for identifying and proceeding with suspected germline variants need to be established in analogy to existing guidelines for somatic tumor tissue analyses. For tumor-only tissue sequencing, the European Society of Medical Oncology (ESMO) recommends reporting of suspected germline variants detected by NGS above a threshold of >30 % VAF for SNVs and >20 % VAF for InDels [55]. The National Comprehensive Cancer Network (NCCN) recommends referral for confirmatory germline testing if an identified tumor variant has clinical implications [56]. Previous studies investigating the incidental detection of germline variants in cfDNA have suggested that heterozygous germline variants are likely to be detected with a VAF of ~50 %, above the VAFs observed for most somatic variants [57]. Recent studies confirmed these results through confirmatory germline testing of variants identified in cfDNA [58], further supporting the suggestion that a VAF-based threshold for identifying suspected germline variants in cfDNA analogous to tissue testing may be critical to ensure the proper interpretation of liquid biopsy analysis results. To enable a VAF based threshold for suspected germline variants also, the overall estimate of the ctDNA fraction identified in a patient should be considered during clinical interpretation of variants identified from liquid profiling. High VAFs of mutations at low tumor fractions (<10 %) indicate a germline origin. However, in late-stage cancer patients, high ctDNA fractions have been described (up to 90 %) [59] and in these cases VAFs alone cannot be used to distinguish germline from somatic variants. Also, low VAF variants at high tumor fractions may indicate either a subclonal origin or clonal hematopoiesis.

Given the clinical implications of incidentally detected germline variants based on cfDNA analysis, the need for interdisciplinary molecular tumor boards and referral to genetic counseling of affected patients is evident. Therefore, informed consent of patients undergoing cfDNA analysis addressing the possibility of incidental findings is required in advance. Patients must be informed about the possibility of detecting incidental findings prior to testing and in case a putative actionable germline variant is identified, this must be confirmed with follow-up germline testing accompanied by expert genetic counseling to enable risk-reducing measures and familial screening. In addition, identified germline variants may facilitate the eligibility for genomically stratified clinical trials or the treatment with molecularly targeted therapies [60].

The involvement of medical geneticists in molecular tumor boards (MTBs) would therefore be helpful to assist oncologists and pathologists in interpreting variants, especially regarding VAF, to discuss germline variants and their consequences, extending the perspective of the expert setting. At the same time, geneticists must also expand their expertise to somatic variant interpretation and possible therapeutic implications to actively participate in the evolving field of molecular medicine.

## Challenges for implementing liquid biopsy into routine clinical practice

As described above, liquid biopsy is on the verge to be introduced into routine clinical practice to support precision medicine in cancer and mosaic disorders. However, especially for highly sensitive and specific detection of variants with lowest VAFs, standardization of diagnostic workflows is indispensable. Already pre-analytical variability, such as differences in blood collection tubes (BCTs), transportation, storage conditions and cfDNA isolation, can reduce the cfDNA quality and ultimately lead to disparate results across laboratories. Moreover, differences in the analytical techniques, including the library preparation, sequencing platform and bioinformatics pipeline can introduce bias in data interpretation [61]. To address these challenges and work towards the standardization of liquid biopsy with a global perspective, the international liquid biopsy standardization alliance (ILSA) was founded, comprising a number of organizations such as the BloodPAC consortium and the European liquid biopsy society (ELBS) [62]. Recommendations for the validation of ctDNA NGS assays, developed by the BloodPAC consortium represent an important step in overcoming the lack of standardization and ensure reliable and comparable results enabling meaningful clinical decision-making [63].

With improvements in standardization and quality control of liquid biopsy approaches ctDNA analysis was included into clinical guidelines such as the ESMO guidelines for ctDNA analysis in patients with cancer [5]. Still, health insurances in the DACH-region (Germany, Austria and Switzerland) only reimburse the costs for the corresponding tests in particular cases, and even then, only to a minimal extent, limiting wide access to patients. Reimbursement decisions are typically based on the demonstration of clinical utility, cost-effectiveness, and the ability to improve patient outcomes. To this end, well-designed clinical trials are currently being planned and conducted to demonstrate the clinical validity and utility of liquid biopsy approaches in specific indications, thereby providing sufficient evidence for reimbursement by health insurances. Further, collaborative efforts between researchers, industry stakeholders, regulatory bodies, and patients are essential to navigate the complex landscape of reimbursement and ensure that liquid biopsy-based diagnostics become an integral part of personalized medicine.

## Conclusions

Liquid biopsy is a promising tool for the clinical diagnosis of various diseases, not limited to its current main application in cancer diagnostics. In addition to a better representation of genetic heterogeneity, the minimal invasiveness of liquid biopsy is a crucial advantage over a tissue biopsy. Using state-of-the-art NGS technologies with high sensitivity allows broad genomic coverage of genes and regions of interest. For the correct interpretation of a detected variant, determining the origin of that variant is critical. For example, when liquid biopsy is used for cancer diagnosis, pathogenic germline variants with clinical implications may be detected in addition to tumor variants. Hence, introducing guidelines for confirmation, reporting, and genetic counseling after discovering such variants is essential. Moreover, in a tumor-agnostic NGS analysis approach, it is possible to find typical tumor variants that are not released by the tumor but rather caused by other somatic events such as CHIP. Like incidental findings of germline variants, guidelines for confirmatory testing candidate CHIP variants must be introduced when liquid biopsy is applied in clinical practice. Considering the requirements for clinical interpretation of liquid biopsy results, currently used cfDNA analyses allow reliable CDx in cancer patients and even improve the diagnostic yield of mosaic disorders.

In summary, cfDNA analysis involves multiple aspects of human genetics. In addition to the importance of correct variant interpretation in tumor patients, liquid biopsy also offers the possibility to diagnose the molecular cause of monogenic diseases that may be missed by germline testing. Finally, novel liquid biopsy methods for specific applications are in active development and will emerge shortly to expand the repertoire of clinical questions that can be addressed. An increasing understanding of the underlying biology of DNA release into the circulation and improved methods to interpret the signals, such as the aforementioned “fragmentomics”, will pave the way for such novel liquid biopsy applications, which will likely have a tremendous impact on many areas of medicine. Therefore, liquid biopsies must become part of the training of human geneticists because the spectrum of these techniques will be used routinely for a growing number of questions in the near future.
